# Dosimetric Evaluation of MR-Guided IMRT and VMAT Techniques for Early-Stage Glottic Cancer: A Pilot Comparative Planning Study

**DOI:** 10.3390/medicina62081507

**Published:** 2026-08-05

**Authors:** Viktoras Rudžianskas, Evelina Jaselskė, Jurgita Laurikaitienė, Vita Šimonytė-Verbickienė, Diana Adlienė

**Affiliations:** 1 Oncology Institute, Lithuanian University of Health Sciences, Eiveniu str. 2, LT-50161 Kaunas, Lithuania; viktoras.rudzianskas@lsmu.lt (V.R.); vita.simonyteverbickiene@lsmu.lt (V.Š.-V.); 2 Physics Department, Kaunas University of Technology, Studentu str. 50, LT-51368 Kaunas, Lithuania; jurgita.laurikaitiene@ktu.lt (J.L.); diana.adliene@ktu.lt (D.A.)

**Keywords:** glottic cancer, MR-guided radiotherapy, MR-linac, stereotactic ablative radiotherapy, individual treatment planning, organ at risk sparing, laryngeal motion control, dosimetric statistics

## Abstract

*Background and Objectives*: Early-stage glottic cancer requires highly precise radiotherapy due to laryngeal motion and the proximity of critical structures. Magnetic resonance-guided radiotherapy (MRgRT) enables real-time visualization and motion management, potentially improving dose conformity while reducing treatment margins. This pilot comparative planning study compared the dosimetric characteristics of MR-guided intensity-modulated radiotherapy (IMRT) delivered on the Unity MR-linac with volumetric modulated arc therapy (VMAT) plans generated for the TrueBeam STx and Halcyon systems. *Materials and Methods*: Ten patients with T1–T2N0 glottic squamous cell carcinoma were included. For each patient, treatment plans prescribing 42.5 Gy in five fractions were generated using all three systems. Planning target volume (PTV) coverage, conformity index (CI), homogeneity index (HI), organ-at-risk (OAR) doses, monitor units, and beam-on times were compared using Friedman and post hoc Wilcoxon signed-rank tests. *Results*: Unity achieved superior dose conformity (CI 0.97 ± 0.01 vs. 0.78 ± 0.15 for TrueBeam STx and 0.58 ± 0.19 for Halcyon; *p* < 0.001) and the highest mean dose, while PTV coverage (D95% and D98%) was comparable to TrueBeam STx and higher than Halcyon. Reduced treatment margins resulted in significantly smaller PTV volumes (11.09 ± 3.87 cm^3^ vs. 17.43 ± 4.75 cm^3^; *p* = 0.0001). Halcyon demonstrated the most homogeneous dose distribution (HI 7.88 ± 3.14), whereas TrueBeam STx showed intermediate homogeneity (HI 12.81 ± 3.90). Most OAR dose metrics were comparable among techniques, although selected laryngeal structures and the esophagus differed significantly. Beam-on time was longest for Unity. *Conclusions*: MR-guided IMRT provided superior dose conformity while maintaining target coverage comparable to TrueBeam STx with clinically acceptable OAR sparing. Prospective clinical studies are required to determine whether these dosimetric advantages translate into improved patient outcomes.

## 1. Introduction

Laryngeal tumors most commonly arise at the glottic level, accounting for approximately 50–60% of all laryngeal cancers and are typically diagnosed at an early stage (T1–2N0) [1]. Early-stage glottic cancer may be treated either surgically (open or endoscopic approaches) or with radiotherapy. Both treatment modalities have demonstrated comparable efficacy, with reported overall survival rates of approximately 90% for radiotherapy and 80% for surgical treatment [2,3].

In recent years, increasing attention has shifted from conventional whole-larynx external beam radiotherapy toward localized irradiation of the involved vocal cord, with the aim of minimizing treatment-related toxicity. Clinical outcomes reported in studies investigating single vocal cord irradiation have been shown to be comparable to those achieved with conventional whole-larynx radiotherapy [4,5,6,7].

Stereotactic ablative radiotherapy (SABR) has also emerged as a potential treatment option for early-stage glottic cancer. Sher et al. reported favorable results from a phase II trial in which patients with T1–T2 glottic tumors were treated with 42.5 Gy delivered in five fractions using the CyberKnife system in combination with a swallow-gating protocol managed by surface-guided radiotherapy (SGRT) [8]. In another study, Sanguineti et al. treated patients with T1 glottic tumors using 36 Gy in three fractions delivered to the histologically confirmed involved portion of the true vocal cord (CTV36), and 30 Gy in three fractions to the anterior and posterior regions (CTV30), using two to four arcs of 6 MV flattening filter-free (FFF) photons on a Varian TrueBeam linear accelerator equipped with a 120-leaf Millennium multileaf collimator (MLC) [1]. Although this approach achieved high rates of local tumor control, treatment was discontinued due to an increased incidence of radiation-induced necrosis.

Taken together, accumulating evidence indicates that stereotactic ablative radiotherapy represents a promising treatment strategy for early-stage glottic cancer; however, accurate characterization and management of intrafraction laryngeal motion remains essential for safe and precise dose delivery. Within this context, the present study evaluated treatment plans of patients treated on MR-guided linear accelerator (Unity) and compared them with corresponding virtual treatment plans generated for the TrueBeam STx and Halcyon systems. These three platforms were selected because they represent distinct radiotherapy delivery technologies currently used in clinical practice, including an MR-guided linear accelerator (Unity), a conventional C-arm linear accelerator (TrueBeam STx), and a ring-gantry linear accelerator (Halcyon). We therefore hypothesized that MR-guided radiotherapy combined with comprehensive motion management (CMM) may enhance treatment precision, reduce radiation-induced toxicity, and enable individualized definition of gross tumor volume (GTV)-to-planning target volume (PTV) margins.

The aim of this pilot comparative planning study was to compare the dosimetric characteristics of MR-guided IMRT delivered on the Unity MR-linac system with VMAT plans generated for the TrueBeam STx and Halcyon platforms in patients with early-stage glottic squamous cell carcinoma.

## 2. Materials and Methods

### 2.1. Patient Selection and Simulation

This study was approved by the institutional review board of the Hospital of Lithuanian University of Health Sciences Kaunas Clinics. All patients provided written informed consent prior to enrollment, in accordance with the Declaration of Helsinki. Ten patients diagnosed with early-stage (T1–2N0) glottic squamous cell carcinoma and treated using the Unity MR-linac system (Elekta AB, Stockholm, Sweden) were included in the analysis. Patient characteristics are summarized in Table 1.

All patients underwent both computed tomography (CT) and magnetic resonance imaging (MRI) for treatment planning. CT scans were performed using a LightSpeed RT16 scanner (GE Healthcare, Chicago, IL, USA), and MRI was performed on integrated Unity 1.5 T MR-linac system with Philips Marlin 5.7 software (Elekta AB, Stockholm, Sweden; Philips Healthcare, Best, The Netherlands).. The slice thickness for both imaging modalities was 1.25 mm. Patients were immobilized in the supine position using an MR-compatible head-and-neck baseplate, a headrest, and a standard five-point thermoplastic mask.

### 2.2. Treatment Planning of IMRT Using MR-Linac Unity System

The gross tumor volume (GTV) was delineated based on clinical examination and imaging findings. A clinical target volume (CTV) was not defined. For early-stage glottic cancer, the macroscopic disease is typically confined to the involved true vocal cord, and several contemporary studies have demonstrated that vocal cord-only or individualized partial larynx radiotherapy can achieve excellent local control while reducing the dose to uninvolved laryngeal structures [5,9]. Accordingly, treatment volumes have progressively evolved toward more focal irradiation strategies while maintaining oncological outcomes. In this planning study, direct GTV-to-PTV expansion was used within a highly conformal MR-guided treatment approach. The planning target volume (PTV) was generated by expanding the GTV by 3 mm in all directions, except for a 5 mm expansion in the craniocaudal direction. These asymmetric margins were selected based on published analyses of laryngeal motion demonstrating greater intrafraction displacement in the craniocaudal direction, supporting the use of anisotropic PTV margins [10,11]. All patients received a total dose of 42.5 Gy delivered in five fractions administered twice a week [8].

Organs at risk (OARs), including the arytenoid cartilages, bilateral carotid arteries, inferior pharyngeal constrictor muscle, and other relevant structures, were contoured. Dose constraints for OARs are presented in Table 2 [12,13,14,15]. The thyroid cartilage was selected as the primary registration structure for target localization during intrafraction motion monitoring. Before each treatment fraction, a high-resolution T2-weighted MRI was acquired for patient positioning and online adaptive radiotherapy. All patients were treated using an online adaptive workflow before every fraction. Patients were immobilized using a thermoplastic head-and-neck mask and positioned according to the same external reference marks throughout the treatment course. Intrafraction motion was managed using the Elekta Unity Comprehensive Motion Management (CMM) system (Elekta AB, Stockholm, Sweden), which performs real-time template-based registration of predefined tracking structures and automatically interrupts beam delivery when motion exceeds the predefined tolerance during real-time cine MRI monitoring. Treatment resumed only after satisfactory anatomical alignment had been re-established. Daily MRI was also evaluated for clinically relevant anatomical changes, including tumor regression or reduction in neck volume associated with weight loss. In our institutional clinical workflow, online adaptive replanning was considered when clinically relevant anatomical changes, typically including neck contour changes of approximately 1 cm or greater, were observed. The decision to proceed with replanning was made by the treating radiation oncologist [16,17].

IMRT treatment plans for patients were generated using the Monaco Treatment Planning System version 6.2.2 (Elekta AB, Stockholm, Sweden), GPUMCD Photon dose calculation algorithm, 7MV FFF photon beam and 160 MLCs with longitudinal leaf motion. For all plans 13 beams with predefined gantry angles were used, each focused on the center of treatment target.

Treatment plans were optimized to meet the dosimetric requirements based on carefully selected planning guidelines. The number of segments varied from 31 to 91 (mean 68.7 segment). All parameters for treatment plans were defined as following: grid spacing 0.25 cm, min. segment area 2 cm^2^, min. segment width 0.75 cm, min MU/segment—4.00 and max segments per plan 110.

After plan preparation, the calculated doses and required contoured structures (reference plan) were imported to the MOSAIQ oncology information system (Elekta AB, Stockholm, Sweden) for later online adaptive treatment.

### 2.3. Virtual Treatment Planning of VMAT Using TrueBeam STx and Halcyon

For comparative purposes, VMAT plans were generated using the same CT and MRI datasets as those used for the MR-linac IMRT plans. The GTV definition was identical. The PTV was created by expanding the GTV by 5 mm in all directions, except for an 8 mm expansion in the craniocaudal direction to account for laryngeal motion and setup uncertainties [18]. Larger margins were applied to represent a conventional IGRT-based workflow without real-time MR cine imaging. Published cine-MRI and organ-motion studies have demonstrated greater cranio-caudal laryngeal motion and support the use of larger margins when real-time motion monitoring is unavailable [10,11]. The prescribed dose was 42.5 Gy delivered with the Varian TrueBeam STx linear accelerator (Varian Medical Systems, Palo Alto, CA, USA) in five fractions—8.5 Gy per fraction. All plans were created using Eclipse Treatment Planning System version 16.6 (Varian Medical Systems, Palo Alto, CA, USA) following these parameters: one full (181 > 179 degrees) arc, 6MV FFF beam with a high-definition 120-leaf MLC™ (Varian Medical Systems, Palo Alto, CA, USA). All VMAT plans were optimized using Anisotropic Analytical Algorithm (AAA) algorithm and a 1.25 mm dose calculation grid resolution. After calculation, all treatment plans were normalized to cover the required PTV volume according to the same clinical goals used for laryngeal cancer SBRT treatment plans in MR Unity linac.

### 2.4. Evaluation of the Treatment Plans

For IMRT plans generated using the Unity MR-linac system, the total number of segments, monitor units (MUs), and calculated beam-on time were recorded. For each PTV, minimum, mean, and maximum doses were calculated, along with D2%, D5%, D95%, and D98%. Plan quality was assessed using the homogeneity index (HI) and conformity index (CI). The HI was calculated according to the one equation proposed by Wu et al. [19]:

(1) $$HI=\frac{D_{2}-D_{98}}{D_{p}}\times 100,$$

where D_2_ and D_98_ represent the minimum doses received by 2% and 98% of the target, respectively, and D_p_ is the prescribed dose [19]. Lower HI values indicate greater dose homogeneity within the target volume.

Dose conformity was evaluated using the conformity number (CN) described by van’t Riet et al., referred to as the CI in this study (Equation (2)):

(2) $$CN=\frac{{\left({TV}_{RI}\right)}^{2}}{TV\times V_{RI}},$$

where TV_RI_ is the target volume covered by the reference isodose, TV is the total target volume, and V_RI_ is the volume of the reference isodose [20]. CI values range from 0 to 1, with a value of 1 indicating optimal conformity characterized by complete target coverage without irradiation of surrounding tissues. Values below 1 indicate reduced conformity due to either incomplete target coverage or unnecessary irradiation of adjacent normal tissues.

For organs at risk (OARs), the maximum doses to the contralateral and ipsilateral arytenoid cartilage, contralateral and ipsilateral carotid arteries, skin, esophagus, trachea and thyroid cartilage were calculated. The mean doses to the contralateral and ipsilateral carotid arteries, pharyngeal constrictor muscle and cricoid cartilage were calculated. The percent volume received at least 32.5 Gy (V32.5 Gy) for the esophagus were also calculated. For both the ipsilateral and contralateral arytenoid cartilage D0.1 cc, skin D10 cc, trachea D5 cc, spinal cord D0.03 cc and D0.35 cc doses were calculated.

Statistical analyses were performed using *R* statistical software version R4.5.3 (*R* Foundation for Statistical Computing, Vienna, Austria). Non-parametric methods were used due to the small sample size and the repeated-measures design of the study. Differences among the three radiotherapy techniques (MR-linac Unity, TrueBeam STx, and Halcyon) were evaluated using the Friedman test for the related samples. This test was selected due to the ordinal nature of some parameters and to avoid assumptions of normal distribution across treatment groups. The test statistic *χ*^2^ with two degrees of freedom (*df* = 2) was calculated for each parameter, with significance determined at *α* = 0.05.

When the Friedman test demonstrated statistical significance, post hoc pairwise comparisons were performed using Wilcoxon signed-rank tests with Holm correction for multiple comparisons. To control for multiple testing across all evaluated parameters, Benjamini–Hochberg correction was additionally applied to the Friedman test *p*-values. Data are presented as medians with 95% confidence intervals.

Effect sizes for the Friedman analysis were calculated using Kendall’s coefficient of concordance (Kendall’s *W*) and interpreted as follows: *W* < 0.1, negligible; 0.1–0.3, small; 0.3–0.5, moderate; and >0.5, large effect size. Continuous variables were reported as median and interquartile range (IQR). A two-sided *p*-value < 0.05 was considered statistically significant.

## 3. Results

### 3.1. Differences in the Plan Parameters

**Figure 1:**
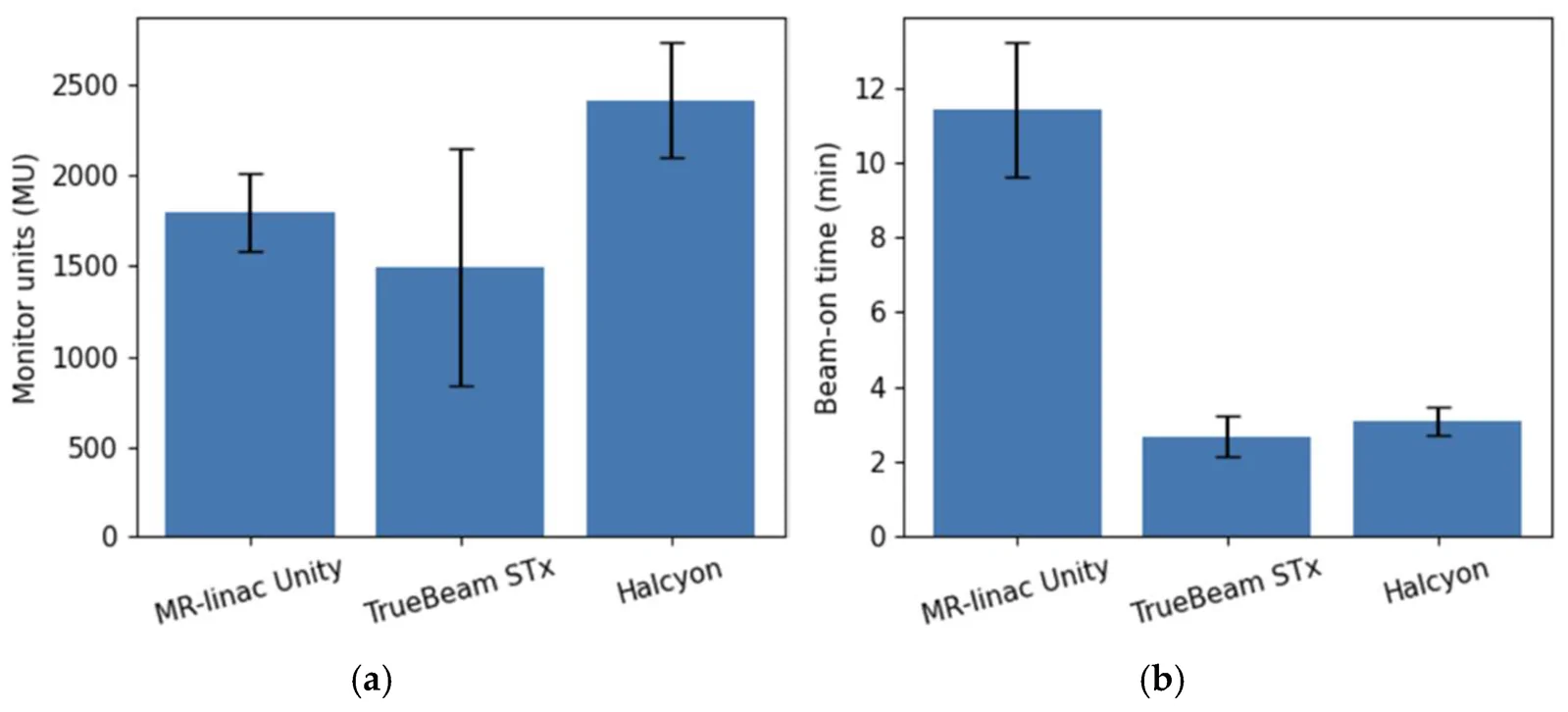
Comparison of treatment plan parameters among IMRT using the MR-linac Unity system and VMAT using the TrueBeam STx and Halcyon systems. (**a**) Monitor units (MUs), (**b**) calculated beam-on time. Error bars represent standard deviation.

The plan parameters of IMRT (Mr-linac Unity) and VMAT (TrueBeam STx and Halcyon) are summarized in Table 3 and illustrated in Figure 1.

A graphical representation was used to facilitate comparison of monitor units and beam-on time across treatment techniques. Figure 1 demonstrates substantial differences in monitor units (MU) and calculated beam-on time among the evaluated treatment techniques. The highest MU values were observed for VMAT using the Halcyon system (2415.8 ± 319.15 MU), whereas the lowest MU values were achieved with VMAT using the TrueBeam system (1488.97 ± 653.54 MU). IMRT plans generated on the Unity MR-linac demonstrated intermediate MU values (1797.62 ± 216.3 MU).

The longest calculated beam-on time was observed for the MR-linac IMRT plans (11.4 ± 1.8 min), while substantially shorter beam-on times were achieved using VMAT techniques. Among the VMAT systems, the shortest beam-on time was obtained with TrueBeam STx (2.69 ± 0.54 min), followed by Halcyon (3.10 ± 0.38 min).

### 3.2. Dose-Volumetric Parameters of PTV

**Figure 2:**
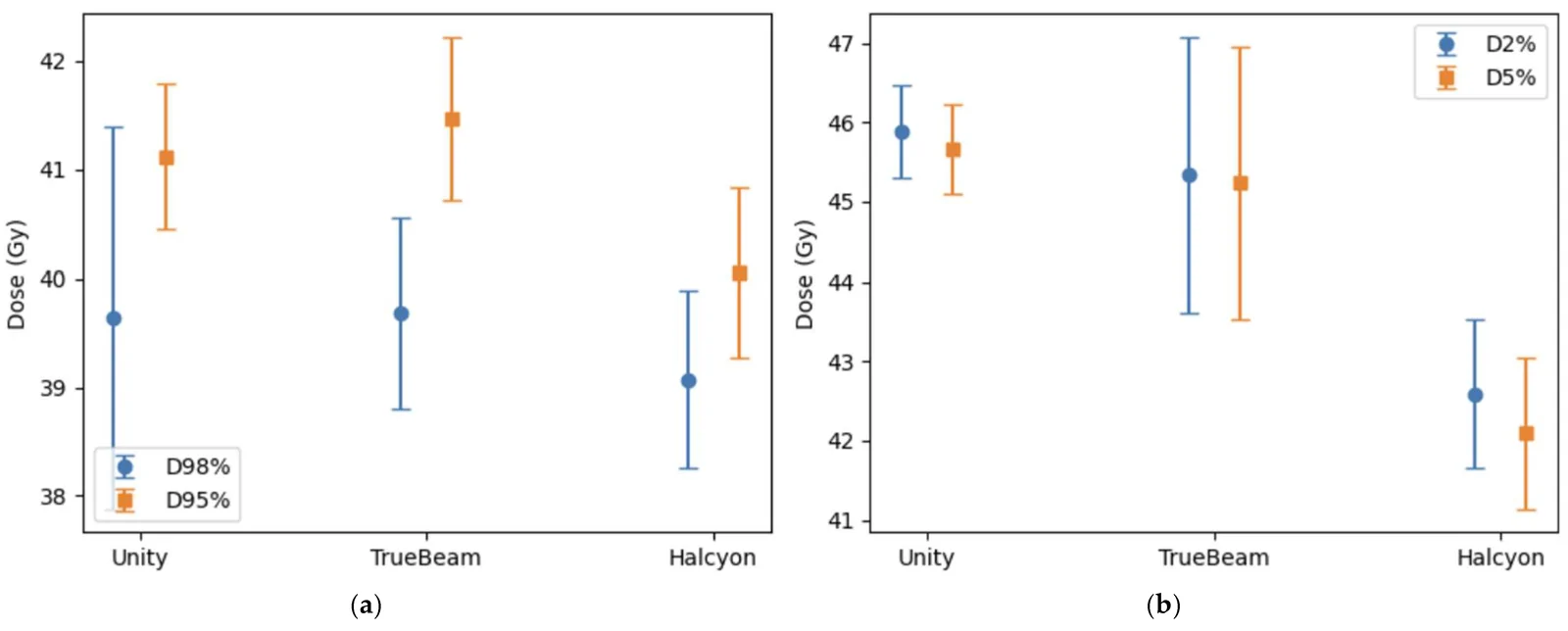
Comparison of dose-volumetric PTV parameters among IMRT using the Unity system and VMAT using the TrueBeam and Halcyon systems. (**a**) PTV coverage parameters (D98% and D95%). (**b**) PTV hotspot parameters (D2% and D5%). Error bars represent standard deviation.

Dose-volumetric (DV) parameters of PTVs for IMRT using the MR-linac Unity system and VMAT using the TrueBeam STx and Halcyon systems are presented in Table 4 and in Figure 2.

Figure 2 demonstrates differences in PTV coverage and hotspot dose parameters among the evaluated treatment techniques. The highest target coverage values (D98% and D95%) were observed for IMRT using the Unity and VMAT using the TrueBeam systems, whereas the lowest values were obtained with Halcyon plans.

For hotspot-related parameters, IMRT plans generated on the Unity MR-linac demonstrated the highest D2% and D5% values, indicating greater dose heterogeneity within the PTV. The TrueBeam STx VMAT plans showed slightly lower hotspot doses than Unity, whereas the Halcyon VMAT plans achieved the lowest hotspot doses among the evaluated techniques.

A statistically significant difference in homogeneity index (HI) was observed among the three treatment delivery systems (Friedman test: χ^2^ (2) = 16.2, *p* = 0.0003; Benjamini–Hochberg corrected *p* = 0.0012), with a large effect size (Kendall’s W = 0.81) indicating clinically meaningful differences in dose distribution uniformity. The Halcyon system demonstrated the most favorable dose homogeneity with the lowest median HI values of 5.70 (5.70 [5.31–8.20]), indicating superior dose uniformity within the planning target volume (PTV). The TrueBeam STx demonstrated intermediate HI values, whereas the MR-linac Unity exhibited the highest HI values, indicating greater dose heterogeneity. Post hoc pairwise analysis using Holm correction revealed statistically significant differences between all three treatment modalities (Unity versus TrueBeam: *p* = 0.0078; Unity versus Halcyon: *p* = 0.0078; TrueBeam versus Halcyon: *p* = 0.0059). These results demonstrate that the treatment delivery modality influences dose uniformity, with implications for clinical site selection and treatment planning optimization.

A statistically significant difference in conformity index (CI) was observed among the three treatment platforms (Friedman test: χ2(2) = 15.8, *p* = 0.0004; Benjamini–Hochberg corrected *p* = 0.0012), with a large effect size (Kendall’s W = 0.79) indicating the substantial influence of treatment modality on dose conformity. The MR-linac Unity system demonstrated superior dose conformity with a median CI of 0.98 (0.98 [0.98–0.99]), significantly outperforming both TrueBeam STx and Halcyon in pairwise post hoc analysis (Holm-corrected *p* = 0.0059 for both comparisons). This superior conformity metric reflects the technological advantages of MR-linac in delivering highly conformal dose distributions, consistent with previous dosimetric evaluations demonstrating enhanced conformity capabilities of MR-guided systems [21]. Notably, no statistically significant difference was detected between the two VMAT-based systems (TrueBeam STx versus Halcyon; *p* = 0.2324), suggesting comparable conformity performance between conventional and ring gantry VMAT platforms despite differences in other dosimetric parameters. The distinction between MR-linac and VMAT-based systems underscores the importance of considering both target coverage accuracy and radiation modality when optimizing treatment plans for various clinical scenarios.

Statistically significant differences in PTV coverage parameters were observed among the three treatment systems. Friedman non-parametric analysis demonstrated significant effects across multiple dose-volume metrics including D2, D5, D95, D98, maximum, mean, and minimum doses (all *p* < 0.05), with predominantly large effect sizes (Kendall’s W = 0.61–0.91) indicating robust and clinically relevant differences between techniques.

The TrueBeam STx achieved the highest target coverage metrics for D95 and D98, whereas the MR-linac Unity demonstrated the highest maximum and mean doses while maintaining superior dose conformity throughout the target volume (CI = 0.97). In contrast, the Halcyon system demonstrated the lowest hotspot doses (D2, D5, and maximum dose) alongside the best homogeneity index values, confirming its capacity to deliver a more homogeneous dose distribution within the target. The TrueBeam STx system demonstrated target coverage comparable to the Unity MR-linac while exhibiting intermediate hotspot dose parameters and homogeneity between Unity and Halcyon.

Pairwise post hoc analysis using Holm correction revealed significant differences between the MR-linac Unity and both VMAT systems across most measured parameters. Additionally, Halcyon and TrueBeam STx differed significantly in several hotspot-related parameters (D2, D5, and maximum dose). Pairwise comparisons also demonstrated that the Unity MR-linac and TrueBeam STx achieved similarly high target coverage for D95 and D98, whereas Halcyon showed lower values for these parameters. This finding is clinically important, indicating that both systems provide excellent target dose coverage while differing in dose homogeneity and conformity characteristics.

Therefore, the results indicate that MR-linac Unity provides superior target coverage and dose conformity, with an excellent ability to deliver prescribed doses to the planning target volume while maintaining tight dose margins. Halcyon achieves the most homogeneous dose distribution with reduced hotspots, making it an optimal choice for clinical scenarios where dose uniformity is prioritized. TrueBeam STx provides target coverage comparable to the Unity MR-linac while demonstrating intermediate dose homogeneity between Unity and Halcyon, representing a balanced compromise between target coverage and dose uniformity.

### 3.3. Dose-Volumetric Parameters of OARs

Dose-volumetric parameters of OARs for IMRT using the MR-linac Unity system and VMAT using the TrueBeam STx and Halcyon systems are presented in Table 5.

**Figure 3:**
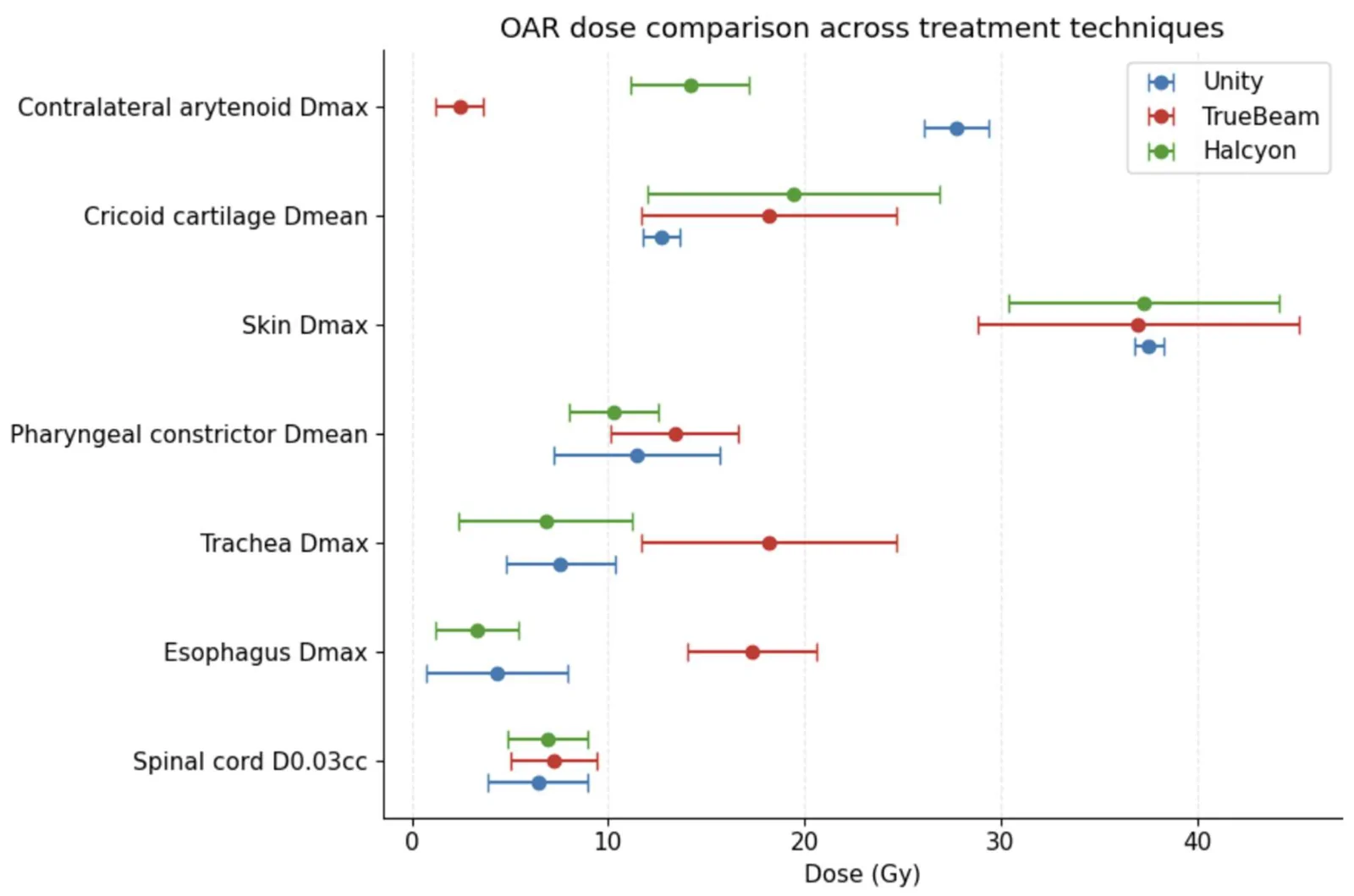
Comparison of organs at risk (OAR) dose metrics among MR-linac and VMAT-based treatment techniques.

To visualize and compare the differences in organ at risk (OAR) dose metrics across the three treatment techniques, a grouped error-bar plot was generated. Figure 3 demonstrates that the most pronounced deviations were observed in the esophagus, trachea, and contralateral arytenoid, where the largest inter-system variability in dose levels was seen, particularly between MR-linac and VMAT-based plans.

The Unity system demonstrated more balanced OAR dose distribution, with relatively consistent sparing across adjacent laryngeal and non-laryngeal structures. The Halcyon system showed the lowest hotspot-related OAR doses, indicating improved dose homogeneity and efficient suppression of high-dose regions. In contrast, the TrueBeam system exhibited the greatest variability in OAR exposure, with comparatively higher doses in several serial organs, including the esophagus and trachea.

Dosimetric analysis revealed that most of the organ at risk (OAR) dose parameters demonstrated statistically comparable performance across the three treatment techniques evaluated. Specifically, dose to critical structures including carotid artery (both *Dmax* and *Dmean*), oesophageal maximum dose, pharyngeal constrictor mean dose, skin doses, spinal cord D0.035 cm^3^, and tracheal maximum dose all showed negligible to small effect sizes (Kendall’s W ≤ 0.20), indicating minimal clinically meaningful differences among modalities.

Despite this general equivalence, several clinically relevant OAR parameters demonstrated statistically significant differences among treatment modalities. The contralateral arytenoid maximum dose (*Dmax*) emerged as a parameter with robust statistical discrimination, differing significantly among the three treatment techniques (Friedman test: χ^2^(2) = 16.8, *p* = 0.0002; Benjamini–Hochberg corrected *p* = 0.001).

The most pronounced inter-system difference was observed for esophagus V32.5 Gy, which demonstrated a strong statistical effect among OAR parameters (Kendall’s W = 1.0, χ^2^(2) = 20, *p* < 0.001).

Post hoc pairwise analysis revealed that many significant findings were primarily driven by distinct differences between Halcyon and the other treatment systems, with Unity and TrueBeam STx demonstrating greater similarity for several parameters.

## 4. Discussion

In the present study, we compared IMRT plans generated with the Unity MR-linac system with virtual VMAT plans created on the TrueBeam STx and Halcyon systems for early-stage glottic cancer.

A major dosimetric advantage of the MR-linac approach in this study was the ability to use a smaller PTV margin than the VMAT plans. The Unity system supports real-time cine MRI-based monitoring and comprehensive motion management. The GTV expansion to PTV could be reduced to 3 mm in the lateral directions and 5 mm craniocaudally, whereas the VMAT plans required a 5 mm expansion in all directions except for an 8 mm craniocaudal margin to account for laryngeal motion and setup uncertainty. This margin reduction likely contributed to the smaller PTV volume in the MR-linac plans and to the observed reduction in normal tissue irradiation. The smaller PTV volume should also be considered when interpreting the superior target coverage and conformity achieved by the MR-linac plans, as larger target volumes generally make it more challenging to achieve highly conformal dose distributions while respecting organ at risk constraints. Therefore, the unequal PTV margins and the resulting differences in target volume represent an inherent volumetric bias that should be considered when interpreting the comparative plan quality. The present findings are consistent with the concept that MRI-guided radiotherapy may improve geometric precision not only through better soft-tissue visualization, but also by enabling a reduction in treatment margins in appropriately selected patients.

The MR-linac plans also demonstrated favorable target dose characteristics. Compared with both VMAT systems, Unity achieved significantly higher mean dose values together with a markedly better conformity index than both VMAT platforms. Target coverage (D98% and D95%) was comparable between Unity and TrueBeam STx, whereas Halcyon demonstrated lower coverage values. Therefore, the observed dosimetric advantages should not be attributed solely to the beam-delivery characteristics of the MR-linac system, but rather to the combined effect of MR-guided image guidance, motion management, and reduced treatment margins. These findings are consistent with previous studies showing that MR-guided radiotherapy can improve geometric precision and dose delivery accuracy through superior soft-tissue visualization and real-time image guidance [21,22,23,24]. In practical terms, this indicates that, in our cohort, the MR-guided plans delivered the prescribed dose more consistently throughout the target while limiting unnecessary irradiation of surrounding tissues.

At the same time, the hotspot-related metrics revealed distinct dosimetric characteristics across the three platforms. Halcyon achieved the lowest D2, D5, and homogeneity index values, indicating a more homogeneous dose distribution within the PTV, consistent with published comparative analyses demonstrating platform-specific differences in dose distributions [25]. In contrast, Unity exhibited relatively higher hotspot values despite providing improved conformity and target coverage. This finding underscores that superior conformity does not necessarily coincide with the lowest intra-target dose heterogeneity and that platform-specific delivery characteristics should be considered when prioritizing conformity versus homogeneity during treatment planning.

The analysis of organs at risk (OARs) further showed that the three techniques were broadly comparable for most structures, but with clear modality-dependent differences in selected parameters. The most relevant differences involved the contralateral arytenoid, esophagus, thyroid cartilage, trachea, and cricoid cartilage. Halcyon achieved the lowest contralateral arytenoid doses and the lowest mean dose to the contralateral carotid artery, suggesting favorable sparing of some laryngeal substructures. In contrast, Unity demonstrated better tracheal sparing in terms of D5 cm^3^ and preserved a balanced overall OAR dose distribution. These differences likely reflect the distinct beam geometries, arc patterns, and MLC characteristics of the compared platforms.

One notable result was the low esophageal exposure in the Unity and TrueBeam STx plans, contrasted with low but measurable exposure in Halcyon plans. Although the absolute differences were small, this parameter showed one of the more pronounced relative modality effects in the study and illustrates that even subtle variations in beam arrangement can influence low-volume dose deposition in nearby serial organs. Similarly, differences in thyroid cartilage dose may be of particular interest in settings where this structure is used as a surrogate structure for intrafraction motion monitoring. These findings reinforce the value of individualized planning when tumors are close to multiple critical laryngeal structures [26].

From a clinical perspective, these dosimetric findings suggest that MR-guided IMRT may represent a promising approach in situations where maximal target conformity and reduction in dose to adjacent normal tissues are prioritized, for example in patients with anterior commissure involvement, larger motion amplitude, or a strong need to preserve voice and swallowing function. However, the longer beam-on time observed with Unity remains an important practical limitation, as treatment time was substantially longer than with VMAT, and this could affect patient comfort and workflow efficiency. Thus, the choice between MR-guided IMRT and VMAT should be based on the relative importance of precision, organ sparing, treatment time, and technology availability, rather than on the present dosimetric findings alone.

This study has several limitations. First, it was a planning study based on a small cohort of ten patients, which limits statistical power and generalizability. Second, the comparison involved virtual plans rather than delivered treatments, so the clinical impact of the dosimetric differences cannot be directly inferred. Third, the platforms differed not only in delivery technique but also in beam geometry, MLC design, and optimization workflow, which makes attribution of differences to a single factor impossible. In addition, direct GTV-to-PTV expansion without an intermediate CTV represents a study-specific planning approach and should be considered when interpreting the dosimetric findings. The smaller PTV margins and resulting target volume used for Unity represent an important volumetric bias that may have contributed to the superior conformity and target coverage observed in this arm. Finally, because laryngeal motion is dynamic and patient-specific, prospective studies with daily imaging and clinical outcome data are needed to confirm whether the observed dosimetric advantages translate into improved toxicity control and functional preservation.

## 5. Conclusions

This pilot comparative planning study demonstrated that, in this small cohort of early-stage glottic cancer patients, MR-guided IMRT plans achieved higher conformity and improved target coverage compared with both VMAT approaches, while maintaining a clinically acceptable balance of organ at risk sparing. These dosimetric advantages were observed in the context of smaller GTV-to-PTV margins enabled by MR-guided workflow and should therefore be interpreted as the combined effect of image guidance, motion management, and margin reduction rather than beam delivery alone. From a dosimetric perspective, these findings suggest that MR-guided IMRT has the potential to offer advantages in selected early glottic tumors, where precise dose delivery and preservation of laryngeal function are key clinical objectives. Further prospective clinical studies are warranted to determine whether these findings translate into improved patient outcomes, including functional outcomes, reduced treatment-related toxicity, and oncological outcomes.

## Figures and Tables

**Table 1:** Patient characteristics.

Characteristic	N (%)
Age (median, IQR)	68.5 (63–74)
Gender	
Male	10 (100%)
Female	0 (0%)
Stage	
T1a	4 (40%)
T1b	0 (0%)
T2	6 (60%)
Affected vocal cord(s)	
Left	4 (40%)
Right	6 (60%)
Anterior commissure involvement	
Yes	7 (70%)
No	3 (30%)
Smoking history	
≤20 pack-years	6 (60%)
>20 pack-years	4 (40%)
Active smoking (during treatment)	
Yes	3 (30%)
No	7 (70%)

Abbreviation: IQR—interquartile range.

**Table 2:** Clinical goals and dose constraints for OARs (SBRT plan/5 fractions).

Structure	Clinical Goals/Dose Constrains
PTV	D0.03cc <= 110% PD
	D98% >= 93% PD (>=90% PD)
	D2% <= 107% PD
OARs	
Spinal cord	Dmax < 25.3–30 Gy (D0.035cc), V22 < 0.35cc [2,3]
Carotids	Dmax < 25-47 Gy [1]
Esophagus	Dmax < 38 Gy, V32.5 < 5cc [4]
Trachea	Dmax < 50 Gy, V45 < 5cc [4]
Skin	Dmax < 39.5 Gy, V36.5 < 10cc [1]
Arytenoid cartilage:	
ipsilateral	Dmax < 42.3 Gy, Dmean < 26 Gy
contralateral	Dmax < 26 Gy, Dmean < 15 Gy [8]
Inferior pharyngeal constrictor muscle	Dmax < 21 Gy, Dmean < 8.5 Gy [8]
Thyroid cartilage	Dmax < 45 Gy [8]
Cricoid cartilage	Dmax < 43 Gy [8]

Abbreviations: OAR—Organ at risk, cc—Cubic centimeter, Dmax—Maximal dose, Dmean—Mean dose, PTV—Planning target volume, PD—prescribed dose.

**Table 3:** Technical characteristics of the treatment.

	IMRT with MR-Linac Unity	VMAT with TrueBeam STx	VMAT with Halcyon
Number of fields	13 ± 0	one full arc	three full arcs
Number of segments	61 ± 30	N/A	N/A
Monitor units (MUs)	1797.62 ± 216.3	1488.97 ± 653.54	2415.8 ± 319.15
Calculated beam on time (min)	11.4 ± 1.8	2.69 ± 0.54	3.10 ± 0.38

Abbreviation: IMRT—intensity modulated radiation therapy; MR-linac—magnetic resonance image linear accelerator; VMAT—volumetric modulated arc therapy.

**Table 4:** Dose-volumetric parameters of the PTV.

DV Parameter	IMRT with MR-Linac Unity	VMAT with TrueBeam STx	VMAT with Halcyon	* p-Value
D_98%_ (Gy)	39.64 ± 1.76	39.58 ± 0.88	39.07 ± 0.82	0.0031
D_95%_ (Gy)	41.12 ± 0.67	40.47 ± 0.75	40.05 ± 0.78	0.0055
D_5%_ (Gy)	45.66 ± 0.57	44.24 ± 1.71	42.09 ± 0.95	0.0006
D_2%_ (Gy)	45.88 ± 0.58	44.54 ± 1.73	42.59 ± 0.94	0.0004
Minimum dose (Gy)	36.38 ± 1.77	35.52 ± 1.79	31.17 ± 5.62	0.0037
Maximum dose (Gy)	46.64 ± 0.64	45.0 ± 0.14	43.44 ± 1.08	0.0002
Mean dose (Gy)	43.91 ± 0.28	42.58 ± 0.93	41.48 ± 0.77	0.0005
PTV volume (cm^3^)	11.09 ± 3.87	17.43 ± 4.75	17.43 ± 4.75	0.0001
Homogeneity index	14.69 ± 4.8	12.81 ± 3.90	7.88 ± 3.14	0.0004
Conformity index	0.97 ± 0.01	0.78 ± 0.15	0.58 ± 0.19	0.0005

Abbreviation: DV parameter—dose-volumetric parameter; IMRT—intensity modulated radiation therapy; MR-linac—magnetic resonance image linear accelerator; VMAT—volumetric modulated arc therapy; Dn%—dose received by at least n% volume of the planning target volume; PTV—planning target volume. * *p*-values represent the overall comparison among the three treatment planning systems (MR-linac Unity, TrueBeam STx, and Halcyon) using the Friedman test. Pairwise post hoc comparisons were performed using the Wilcoxon signed-rank test with Holm correction, and significant pairwise differences are described in the Results section.

**Table 5:** Dose-volumetric parameters of OARs.

OAR	DV Parameter	IMRT with MR-Linac Unity	VMAT with TrueBeam STx	VMAT with Halcyon	* p-Value
Spinal Cord	D0.03 cm^3^	6.42 ± 2.52	7.22 ± 2.21	6.93 ± 2.02	0.3012
	D0.35 cm^3^	5.33 ± 2.36	6.95 ± 2.12	6.63 ± 1.99	0.0247
Contralateral aryte-	Dmax	27.72 ±1.64	2.42 ± 1.23	14.16 ± 3.04	0.0002
noid	D0.1 cm^3^	20.9 ± 1.52	0.76 ± 0.39	12.27 ± 9.44	0.3012
Ipsilateral arytenoid	Dmax	38.17 ± 5.85	38.79 ± 4.19	36.02 ± 3.96	0.3679
	D0.1 cm^3^	35.69 ± 6.01	15.87 ± 4.26	26.52 ± 6.2	0.0074
Contralateral caro-	Dmax	12.01 ± 2.62	11.04 ± 3.49	11.56 ± 1.99	0.9747
tid	Dmean	5.88 ± 0.86	5.8 ± 1.47	4.69 ± 1.05	0.0366
Ipsilateral carotid	Dmax	15.5 ± 6.33	11.04 ± 3.49	16.08 ±3.18	0.1319
	Dmean	5.69 ± 2.21	5.8 ± 1.47	6.25 ± 1.46	0.1496
Pharyngeal con-	Dmean	11.45 ± 4.24	13.39 ± 3.25	10.29 ± 2.27	0.9747
strictor					
Skin	Dmax	37.55 ± 0.76	37.01 ± 8.16	37.28 ± 6.91	0.7408
	D10 cm^3^	13.46 ± 5.08	2.72 ± 3.13	11.61 ± 4.41	0.2725
Esophagus	V32.5 Gy	0	9.14 ± 4.04	0.84 ± 1.02	<0.0001
	Dmax	4.34 ± 3.57	17.35 ± 3.28	3.34 ± 2.13	0.7939
Trachea	Dmax	7.57 ± 2.8	18.19 ± 6.53	6.82 ± 4.44	0.2725
	D5 cm^3^	0.45 ± 0.03	1.91 ± 0.89	0.62 ± 1.12	0.0247
Thyroid cartilage	Dmax	44.66 ± 1.03	20.07 ± 9.89	41.22 ± 4.35	0.0247
Cricoid cartilage	Dmean	12.73 ± 0.93	18.19 ±6.53	19.46 ± 7.42	0.0208

Abbreviation: *OAR*—organ at risk; *IMRT*—intensity modulated radiation therapy; *MR-linac*—magnetic resonance image linear accelerator; *VMAT*—volumetric modulated arc therapy; *V_nGy_*—percent volume receiving n Gy; *D_ncc_*–minimum dose received by the most irradiated n cm^3^ volume.; *V_nGy_*—percent volume receiving n% of the prescription dose. * *p*-values represent the overall comparison among the three treatment planning systems (MR-linac Unity, TrueBeam STx, and Halcyon) using the Friedman test. Pairwise post hoc comparisons were performed using the Wilcoxon signed-rank test with Holm correction, and significant pairwise differences are described in the Section 3.

## Data Availability

Data is available from authors upon reasonable request.
